# Mucosal and Systemic Immune Responses to *Mycobacterium tuberculosis* Antigen 85A following Its Co-Delivery with CpG, MPLA or LTB to the Lungs in Mice

**DOI:** 10.1371/journal.pone.0063344

**Published:** 2013-05-10

**Authors:** Julie Todoroff, Muriel M. Lemaire, Catherine Fillee, Fabienne Jurion, Jean-Christophe Renauld, Kris Huygen, Rita Vanbever

**Affiliations:** 1 Pharmaceutics and Drug Delivery Group, Louvain Drug Research Institute, Université catholique de Louvain, Brussels, Belgium; 2 de Duve Institute, Experimental Medicine Unit, Université catholique de Louvain, Brussels, Belgium; 3 Ludwig Institute for Cancer Research, Brussels branch, Brussels, Belgium; 4 Department of Clinical Biology, Cliniques Universitaires Saint-Luc, Brussels, Belgium; 5 Service Immunology, Scientific Institute of Public Health (WIV-ISP Site Ukkel), Brussels, Belgium; University of Delhi, India

## Abstract

Pulmonary vaccination is a promising route for immunization against tuberculosis because the lung is the natural site of infection with *Mycobacterium tuberculosis.* Yet, adjuvants with a suitable safety profile need to be found to enhance mucosal immunity to recombinant antigens. The aim of this study was to evaluate the immunogenicity, the safety and the protective efficacy of a subunit vaccine composed of the immunodominant mycolyl-transferase antigen 85A (Ag85A) and one of three powerful mucosal adjuvants: the oligodeoxynucleotide containing unmethylated cytosine-phosphate-guanine motifs (CpG), the monophosphoryl lipid A of *Salmonella minnesota* (MPLA) or the B subunit of heat-labile enterotoxin of *Escherichia coli* (LTB). BALB/c mice were vaccinated in the deep lungs. Our results showed that lung administration of these adjuvants could specifically induce different types of T cell immunity. Both CpG and MPLA induced a Th-1 type immune response with significant antigen-specific IFN-γ production by spleen mononuclear cells *in vitro* and a tendency of increased IFN-γ in the lungs. Moreover, MPLA triggered a Th-17 response reflected by high IL-17A levels in the spleen and lungs. By contrast, LTB promoted a Th-2 biased immune response, with a production of IL-5 but not IFN-γ by spleen mononuclear cells *in vitro*. CpG did not induce inflammation in the lungs while LTB and MPLA showed a transient inflammation including a neutrophil influx one day after pulmonary administration. Pulmonary vaccination with Ag85A without or with MPLA or LTB tended to decrease bacterial counts in the spleen and lungs following a virulent challenge with *M. tuberculosis* H37Rv. In conclusion, CpG and MPLA were found to be potential adjuvants for pulmonary vaccination against tuberculosis, providing Th-1 and Th-17 immune responses and a good safety profile.

## Introduction

One third of the world population is currently infected with *Mycobacterium tuberculosis (M. tuberculosis)* and an estimated 1.4 million people died from tuberculosis (TB) in 2010 [1]. The current bacille Calmette-Guérin vaccine (BCG) has failed to control the TB epidemic. BCG protects children against extrapulmonary TB but shows variable protection in adults against pulmonary TB [2]. Therefore, the development of a new vaccine is important to achieve the goal of the Stop TB Partnership’s Working Group to eradicate the disease by 2050 [3].

Subunit vaccines, based on recombinant proteins admixed with adjuvants or expressed by attenuated viral vectors, are one of the solutions proposed in the search for a new vaccine against TB. Given the protection BCG confers against disseminated disease in childhood, the current view is that subunit vaccines will be mostly used to boost BCG [4]. The most advanced vaccine candidate, now in phase IIb clinical trials, is a recombinant strain of modified Vaccinia virus Ankara expressing immunodominant antigen 85A (Ag85A) from *M. tuberculosis,* delivered by the intradermal route [5]. Antigen 85 is a complex of three proteins, Ag85A, B and C of 30–32 000 Da, which possesses mycolytransferase activity and catalyses the synthesis of a cord factor, an abundant glycolipid of the mycobacterial cell wall [6]. Both Ag85A and Ag85B are immunodominant antigens strongly recognized in humans who control *M. tuberculosis* infection and both have a well documented vaccine potential [7].

Delivery of TB vaccines to the lungs may further increase protection against the disease because it generates the immunity at the natural site of *M. tuberculosis* infection [8]. Other advantages of pulmonary vaccination include: i) the non-invasiveness of the method, which leads to improved patient compliance and to a decreased risk of blood diseases transmission; ii) an activation of both mucosal and systemic adaptive immunity; and iii) a Th-1 biased immune response as compared to injection, especially following vaccine administration to the deep lungs [9]–[11]. Yet, if one would like to consider the pulmonary route for delivery of TB subunit vaccines, one needs to identify safe adjuvants able to properly direct immune responses towards protection.

We evaluated the ability of the potent adjuvants CpG, MPLA or LTB to induce Ag85A-specific immune responses which correlate with protection against TB following pulmonary delivery. All have been shown to induce CD4+ Th-1 and weak CD8+ T cell responses following systemic co-administration with antigen [12]–[14]. Yet, intensity and Th-bias of immune responses depend on the route of administration of the adjuvants, on their recognition of different receptors and activation of different signalling pathways [15]. MPLA triggers toll-like receptor 4 (TLR4) at the surface of alveolar macrophages, dendritic cells, epithelial cells, T and B cells, which in turn stimulates the signalling pathways mediated by the TIR domain-containing adapter inducing interferon-beta [16]. CpG interacts with TLR9 located intracellularly, in the wall of the endosome, mainly in plasmacytoid dendritic cells and B cells. Ligation of TLR9 activates the adapter proteins Myeloid differentiation primary response gene 88 (MyD88) pathway [17]. LTB binds the ganglioside GM1, a glycosphingolipid found ubiquitously on cell surfaces. GM1 mediates LTB presentation by B cells and dendritic cells and LTB presentation enhances the proliferation and cytokine expression of CD4+ T cells [18]. CpG and LTB have already been used in clinical trials while MPLA is included in the composition of two licensed injectable vaccines [19]–[21].

In this study, we co-delivered Ag85A and the adjuvants to the deep lungs in mice and we assessed the intensity and polarization of immune responses to Ag85A: i) in serum and broncho-alveolar lavages (BALs) by analyzing antigen-specific Ig subclasses, ii) in the spleen by using culture of splenocytes restimulated *in vitro* with Ag85A and analysis of their production of cytokines and iii) in the lungs following intratracheal restimulation with Ag85A *in vivo* and analysis of cytokine expression. We also evaluated the safety of the three adjuvants to the lungs by analyzing biochemical and cellular markers of inflammation in BALs. Finally, we evaluated the protective efficacy provided by the subunit vaccines against an intratracheal challenge with a virulent strain of *M. tuberculosis* H37Rv.

## Materials and Methods

### Mice

Specific-pathogen-free female BALB/cJRj mice, aged between 6 and 8 weeks, were used for the experiments (Janvier elevage, Le Genest-St-Isle, France). All experimental procedures were approved by the Institutional Animal Care and Use Committee of the Université catholique de Louvain (Permit number: UCL/MD/2008/039). All studies were performed under anesthesia and all efforts were made to minimize suffering.

### Recombinant Ag85A Protein and I-E^d^ Restricted Synthetic Peptide 11

Hexa-histidine tagged Ag85A protein from *M. tuberculosis* was purified from recombinant *E. coli* as described before [22]. The endotoxin level measured with the Limulus Amebocyte Lysate kinetic chromogenic assay was less than 0.065 EU/µg of purified protein (Lonza Verviers Sprl, Belgium).

The I-E^d^ restricted synthetic peptide 11 was purchased from ProImmune Ltd, Oxford, UK. Peptide 11, spanning amino acid 101 to 120 of Ag85A, is the strongest H-2^d^ restricted CD4^+^ T cell epitope of Ag85A [23].

### Vaccination Protocol

BALB/cJRj mice were anesthetized by intraperitoneal injection of ketamine-xylazine at 75 mg/kg and 8.4 mg/kg, respectively. Then, mice received the vaccine by intratracheal instillation (IT) in the deep lungs [11]. Mice were vaccinated three times at three weeks interval (on days 1, 21, 42) with 5 µg of purified recombinant Ag85A alone or combined with 5 µg CpG C274 (sequence 5′-TCG-TCG-AAC-GTT-CGA-GAT-GAT-3′, Eurogentec S.A, Seraing, Belgium), 5 µg MPLA (Sigma, USA) or 5 µg LTB (Sigma, USA). Control mice were injected 100 µl subcutaneously (SC) in the back, three times at three weeks interval (on days 1, 21, 42), with 5 µg of purified recombinant Ag85A alone or emulsified in Gerbu adjuvant (GERBU Biochemicals, Germany). Gerbu adjuvant is a colloidal suspension composed of biodegradable cationic lipid nanoparticles (octa−/hexadecane) completed with a cell wall subunit of *Lactobacillus bulgaricus* [24]. Other control mice were instilled in the deep lungs with phosphate buffer solution (PBS) or CpG, MPLA or LTB alone.

### Sample Collection

**Figure 1:**
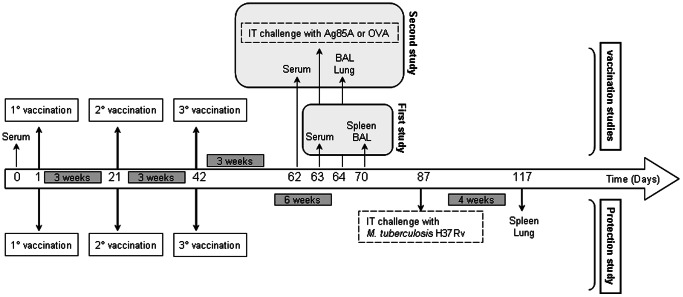
Vaccination and protection study protocols. For the first and second vaccination studies, mice were vaccinated three times at three weeks interval. In the first study, spleen and BAL were collected on day 70, four weeks after the last vaccination. In the second vaccination study, mice were restimulated by intratracheal instillation (IT) with either Ag85A or with OVA three weeks after the last vaccination. One day later, their BAL and lungs were taken for analysis. For the protection study, mice were vaccinated according to the same protocol as for the vaccination studies. Six weeks after the last immunization, mice were challenged intratracheally with luminescent *M. tuberculosis* H37Rv. Bacterial load was quantified in spleen and lungs four weeks post-challenge.

Two studies with the vaccination protocol described above were conducted (Fig. 1). In the first vaccination study, sera were collected from the retro-orbital plexus on day 0 and on day 63 and stored at −20°C until assay. Four weeks after the third vaccination (day 70), mice were killed with an overdose of pentobarbital, spleens were removed aseptically and broncho-alveolar lavages (BALs) were performed with 1 ml Hanks’ Balanced Salt Solution (Sigma, USA), as described before [11]. In the second vaccination study, sera were collected from the retro-orbital plexus on day 0 and on day 62 and stored at −20°C until assay. Three weeks after the third vaccination (day 63), mice were restimulated by intratracheal instillation of 5 µg of Ag85A or 5 µg of ovalbumin (OVA, Grade V, Sigma, USA). One day after the challenge (day 64), mice were killed with an overdose of pentobarbital, BALs were performed and lung samples were collected for reverse transcription-quantitative polymerase chain reaction. BAL samples were concentrated 2.5 times by using SpeedVacPlus SC 110A (ThermoSavant, USA) before performing IL-17A assay by sandwich ELISA using coating antibody MM173G9, biotinylated antibody MM17F3 (Experimental Medicine Unit, UCL, Belgium) and the standard murine recombinant IL-17A (R&D Systems Europe Ltd, Abingdon, UK).

### Antibody ELISA Assays

Ag85A-specific IgG, IgG1, IgG2a were measured in sera and Ag85A-specific IgA and IgG were measured in BALs by ELISA both in the first and second vaccination studies. Specific anti-Ag85A antibodies were determined on serial two-fold dilutions of sera and BALs, using recombinant Ag85A for coating (8 µg/ml), appropriate peroxidase-labelled rat anti-mouse total IgG, IgG1, IgG2a, IgA for detection (IMEX, UCL, Brussels, Belgium) and orthophenyldiamine for revelation (Sigma, USA). Optical densities were read at 492 nm. Antibody titers were defined as the dilution corresponding to an optical density of 0.2 at 492 nm. Individual IgG2a/IgG1 ratios were calculated by dividing individual IgG2a titers by individual IgG1 titers.

### Proliferative Response and Cytokine Production

Splenocytes were adjusted to a concentration of 4×10^6^ white blood cells/ml and were cultivated in 96-well round-bottom microtiter plates (Escolab, Kruibeke, Belgium) in RPMI 1640 medium supplemented with 10% (v/v) fetal calf serum, 0.05 mM 2-mercaptoethanol, 1% (v/v) sodium pyruvate, antibiotics and 1% (v/v) non essential amino acid (MEM, Gibco, Merelbeke, Belgium). Cells were incubated at 37°C in a humidified CO_2_ incubator and stimulated with purified recombinant Ag85A (5 µg/ml), synthetic Ag85A_101–120_ peptide (10 µg/ml) or Concanavalin A (5 µg/ml). Negative control cultures were left unstimulated. Cultures were incubated for 48 h and then pulsed overnight with 0.4 µCi [^3^H] thymidine per well. Cell pellets were filtered onto microplate unifilters and the activity was counted using a TopCount scintillation counter (PerkinElmer, Zaventem, Belgium). Results are expressed in counts per minute (c.p.m). In separate wells, supernatants were collected either after 24 h for interleukin-2 (IL-2) or after 72 h for interleukin-5 (IL-5), interleukin-10 (IL-10), interleukin-17A (IL-17A) and interferon-gamma (IFN-γ) assays (murine IFN-γ ELISA development kit from Peprotech EC LTD, London, UK; IL-2, IL-5 and IL-10 duoset ELISA development kit from R&D Systems Europe Ltd, Abingdon, UK). IL-17A levels were quantified as described above.

### Reverse Transcription-quantitative Polymerase Chain Reaction

Lung samples were collected and stored at −80°C in RNAse free condition. Total RNA was extracted by adding Tripure isolation reagent according to manufacturer’s procedure (Roche, Europe). One microgram RNA was included in reverse transcription with oligo(dT) primers (Roche, Europe) and Moloney murine leukemia virus reverse-transcriptase enzyme (Invitrogen, Europe). cDNA were diluted five times. Quantitative PCR reactions were run in a Thermocycler CFX Real Time System (Biorad, USA) and performed using qPCR Mastermix TaqMan (Eurogentec S.A, Seraing, Belgium), primer pairs and probes specific for murine IFN-γ, IFN-γ-inducible GTPase (Iigp-1), IL-17A, IL-10, β-actin and using qPCR Mastermix SYBR Green 1 (Eurogentec S.A, Seraing, Belgium) and primer pairs specific for IL-5. Reactions were performed at 95°C for 10 min, followed by 40 cycles of 95°C for 15 s, and 60°C (or 61°C for IL-5) for 60 s. For SYBR Green, melting point analysis was carried out by heating the amplicon from 60°C to 95°C. Results were analyzed by MIQ software (Biorad, USA). Transcripts were normalized to the level of the murine β-actin.

### Toxicity Study Protocol

BALB/c mice were anesthetized by intraperitoneal injection of ketamine-xylazine before receiving adjuvant alone by intratracheal instillation in the deep lungs, as described above. Mice were instilled once with 5 µg CpG C274, 5 µg MPLA or 5 µg LTB. Negative and positive control mice were instilled with PBS and 5 µg of lipopolysaccharide (LPS; *Escherichia coli* O111:B4, Sigma, USA), respectively. BALs were collected 4 h, 24 h, 72 h, 7 days and 14 days after intratracheal administration. The lungs were lavaged twice with 1 ml Hanks’ Balanced Salt Solution, as described above. The lavages were then centrifuged (281 g at 4°C for 10 min).

### Biochemical and Cellular Markers of Inflammation

BAL total proteins and lactate dehydrogenase activity (LDH) were spectrophotometrically assayed in the supernatants of the first lavages with the Synchron LX Systems (Beckman Coulter Inc., Brea, USA), as described before [11]. Serum albumin (Imtec Diagnostics N.V., Antwerpen, Belgium) and TNF-α levels (Peprotech EC LTD, London, UK) were measured by ELISA.

The cells of both BAL lavages were pooled. The total number of live cells in the BALs was determined by using the Türk’s solution (VWR International, Leuven, Belgium). The differential cell counts were obtained by cytocentrifugation and coloration with Diff quick (Dade NV/SA).

### Protection Study

Six weeks after the third immunization, mice were challenged intratracheally with 10^3^ mRLU (corresponding to approximately 2×10^3^ CFU) of luminescent *M. tuberculosis* H37Rv [25]. Four weeks after the challenge, mice were euthanized with an overdose of pentobarbital (Fig. 1). The spleen and lungs were homogenized and analyzed by luminometry using a Turner Design 20/20 luminometer and 1% *n-*decanal (Sigma, USA) in ethanol as substrate [26]. The results obtained in milli-relative light units (mRLU) were converted to mean log_10_ mRLU/organ before statistical analysis.

### Statistical Analysis

All results are expressed as mean ± standard error of the mean (SEM). One-way ANOVA and Tukey’s post-test were performed to demonstrate statistical differences using the software GraphPad Prism 5 for Windows. Protective efficacy was analyzed using Student’s *t-*test. Groups of 6 to 8 mice were used for the immunogenicity and toxicity studies. For the second vaccination study, 2 to 3 mice were challenged with OVA and 4 to 5 mice were challenged with Ag85A for each group of 6 to 8 mice. For the protection study, 4 to 5 mice per group were challenged with virulent *M. tuberculosis*. Value of p<0.05 was considered statistically significant.

## Results

### Ag85A-specific Humoral Immune Responses

**Figure 2:**
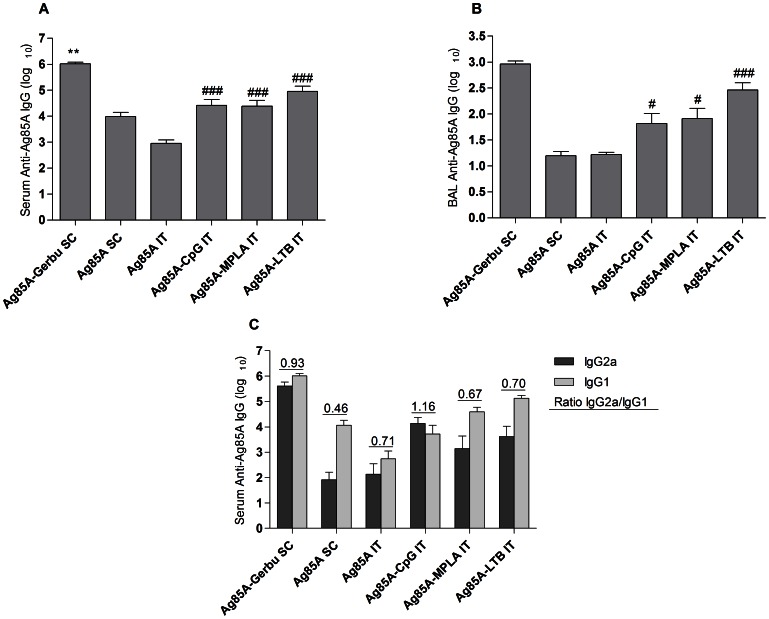
Humoral immune responses in sera and broncho-alveolar lavages (BALs). A. Serum anti-Ag85A IgG titers. B. BAL anti-Ag85A IgG titers. C. Serum anti-Ag85A IgG subclasses titers. SC, subcutaneous injection; IT, intratracheal instillation. * Indicates significant difference from all the other groups. # Indicates significant difference from the group vaccinated in the deep lung with Ag85A alone. One symbol indicates p<0.05. Two symbols indicate p<0.01. Three symbols indicate p<0.001.

All three adjuvants, CpG, MPLA and LTB, enhanced antigen-specific serum IgG titers to Ag85A delivered intratracheally (Fig. 2A). Serum anti-Ag85A IgG reached equivalent levels following pulmonary vaccination with adjuvants and subcutaneous vaccination without adjuvant. Subcutaneous vaccination with Gerbu induced the highest serum IgG titers (Fig. 2A). Antigen-specific IgG titers in broncho-alveolar lavages (BALs) paralleled serum IgG titers, suggesting that serum IgG transudated into the lungs (Fig. 2B). Ag85A-specific antibody titers were below the detection level in the PBS group and in the control groups vaccinated with adjuvants alone and no antigen (data not shown). IgA was assayed in BAL but was undetectable, probably due to the high dilution of BAL samples.

IgG2a and IgG1 were quantified as an indirect assessment of polarization of the T-helper cells population. Pulmonary vaccination without adjuvant generated a higher IgG2a to IgG1 ratio than subcutaneous vaccination without adjuvant (Fig. 2C), indicating a slight shift toward a T helper type 1 phenotype following pulmonary vaccination. Adding CpG to Ag85A further increased IgG2a to IgG1 ratio following pulmonary vaccination, with a ratio reaching over 1, while MPLA and LTB had no impact on this ratio (Fig. 2C). IgG titers presented in Fig. 2 were obtained from the second vaccination study but were identical to IgG titers obtained from the first one (data not shown).

### Ag85A-specific Cellular Immune Responses in the Spleen

**Figure 3:**
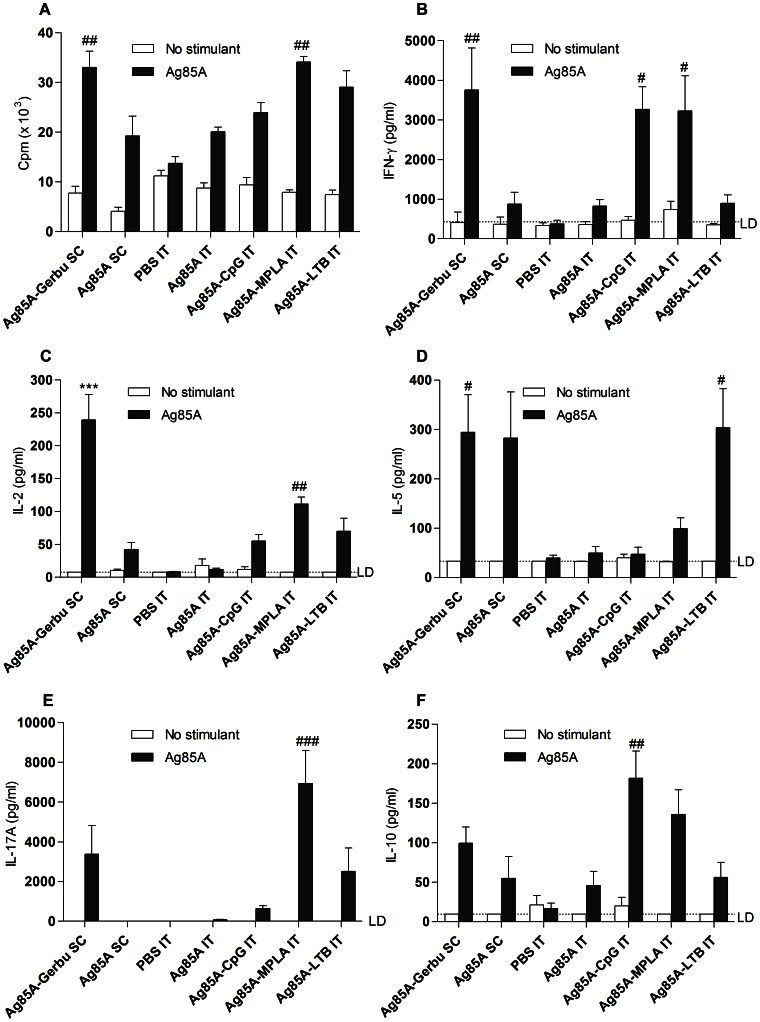
Cellular immune responses in spleen mononuclear cells. A. Proliferation of spleen mononuclear cells following *in vitro* Ag85A stimulation or no stimulation. Results are expressed in counts/minute (cpm). Supernatants from *in vitro* culture of spleen mononuclear cells were assayed for B. IFN-γ, limit of detection (LD) = 429 pg/ml. C. IL-2, LD = 8 pg/ml. D. IL-5, LD = 33.6 pg/ml. E. IL-17A, LD = 8.6 pg/ml F. IL-10, LD = 9.6 pg/ml. SC, subcutaneous injection; IT, intratracheal instillation. * Indicates significant difference from all the other groups. # Indicates significant difference from the group vaccinated in the deep lung with Ag85A alone. One symbol indicates p<0.05. Two symbols indicate p<0.01. Three symbols indicate p<0.001.

Pulmonary administration of Ag85A mixed with all three adjuvants, CpG, MPLA or LTB, enhanced antigen-specific immune responses of splenocytes, as assessed during an *in vitro* recall response (Fig. 3). Pulmonary vaccination with Ag85A and adjuvants induced statistically equivalent splenocyte proliferation *in vitro* as subcutaneous vaccination with Ag85A and Gerbu (Fig. 3A). Yet, only MPLA significantly increased splenocytes proliferation *in vitro* following pulmonary delivery, as compared to pulmonary vaccination without adjuvant (Fig. 3A).

Splenocytes collected from mice vaccinated intratracheally together with CpG or MPLA produced high levels of IFN-γ following *in vitro* restimulation with Ag85A and the IFN-γ levels were comparable to those obtained following subcutaneous vaccination with the potent Th-1/Th-2 Gerbu adjuvant (Fig. 3B). Although formulation in Gerbu induced the highest production of IL-2 by splenocytes, formulation in MPLA significantly increased IL-2 as well. In contrast to IFN-γ production, formulation in CpG induced only low Ag85A specific IL-2 levels. IL-2 levels in Ag85A-LTB immunized animals were also very low (Fig. 3C).

In line with a Th-1 phenotype, splenocytes produced undetectable or very low levels of the Th-2 cytokine, IL-5, following pulmonary vaccination with CpG or MPLA or without adjuvant (Fig. 3D). In contrast, an antigen-specific IL-5 response was detected in spleen cell cultures from mice vaccinated with the pulmonary administered Ag85A in LTB and in both subcutaneously vaccinated groups (Fig. 3D).

Th-17 and regulatory T cells are two other CD4+ T cell subpopulations that play a role in immunity against tuberculosis [27]. MPLA strongly increased spleen cell IL-17A production. There was also some production of IL-17A in the LTB and Gerbu groups (Fig. 3E). Immunization with Ag85A adjuvanted in CpG, MPLA or Gerbu stimulated significant IL-10 production by spleen mononuclear cells while IL-10 production was lower in the LTB group and in the groups vaccinated without adjuvant (Fig. 3F). Cytokine levels were undetectable in the control groups vaccinated with adjuvants alone and no antigen (data not shown). Splenocyte stimulation with the synthetic Ag85A_101–120_ peptide induced the same proliferative response and cytokine profile as the whole Ag85A (data not shown).

### Ag85A-specific Cellular Immune Responses in the Lungs

**Figure 4:**
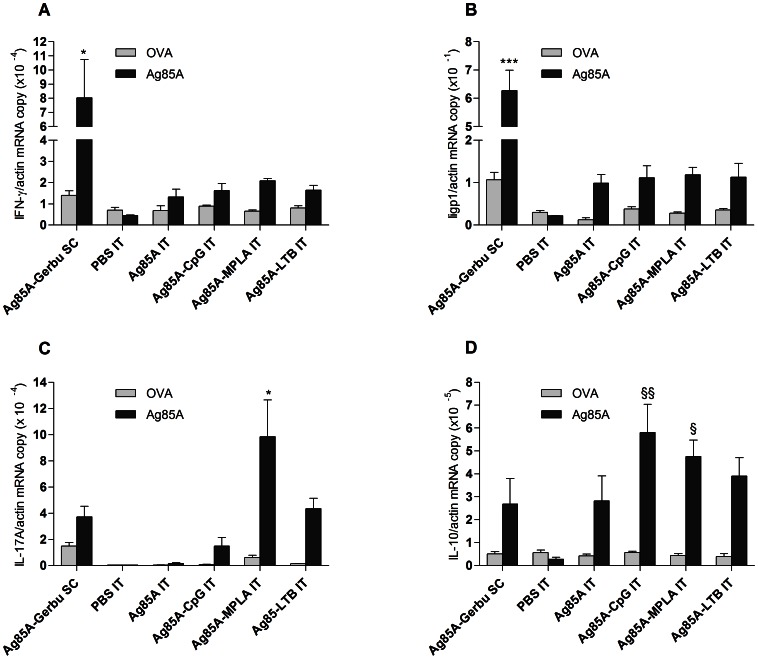
Cellular immune responses in the lungs. Quantitative RT-PCR was performed to measure cytokine mRNA copies in the lungs namely A. IFN-γ. B. Iigp-1. C. IL-17A. D. IL-10. SC, subcutaneous injection; IT, intratracheal instillation. § Indicates significant difference from the PBS group. * Indicates significant difference from all the other groups. One symbol indicates p<0.05. Two symbols indicate p<0.01. Three symbols indicate p<0.001.

Antigen-specific cellular immune responses in the lungs were assessed in mice following intratracheal restimulation with Ag85A or ovalbumin (OVA) (Fig. 1). Restimulation with Ag85A induced IFN-γ, IFN-γ-inducible GTPase gene (Iigp-1), IL-17A and IL-10 expressions whereas OVA did not. Subcutaneous vaccination using Gerbu induced the highest IFN-γ expression in the lungs. Pulmonary vaccination with or without adjuvants produced a trend towards increased IFN-γ expression, as compared to the PBS control group (Fig. 4A). Iigp-1 levels closely followed IFN-γ levels, but the differences with the PBS group were more marked (Fig. 4B). MPLA induced the highest expression of IL-17A (Fig. 4C). Expression of IL-10 increased in all vaccinated groups but expression levels were only significantly increased in the CpG and MPLA groups (Fig. 4D). Overall, expression in the lungs of IL-17A and IL-10 but not of IFN-γ correlated with production of these cytokines in the spleen (Figs. 3–4). IL-5 expression was low for all the groups and no difference was observed between Ag85A or ovalbumin restimulation (data not shown).

IL-17A cytokine production in BALs strongly correlated with IL-17A mRNA expression (Figs. S1, 4C). Among the groups, MPLA combined with Ag85A produced the highest IL-17A protein increase in BAL as it produced the highest IL-17A mRNA expression. Iigp-1 induced downstream by IFN-γ protein highly correlated with IFN-γ mRNA expression (Figs. 4A, 4B), indicating that the production of IFN-γ protein closely followed IFN-γ mRNA expression as well.

### Biochemical Markers of Inflammation Following Pulmonary Delivery of the Adjuvants

**Figure 5:**
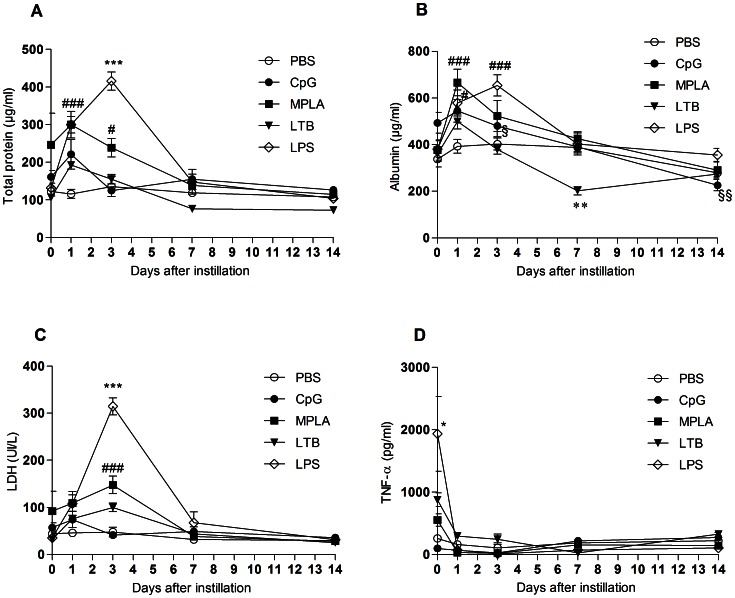
Effect of the adjuvants on inflammatory biochemical markers. A. Total proteins. B. Albumin, LD = 4.4 pg/ml. C. LDH. D. TNF-α levels, LD = 109 pg/ml. # Indicates significant difference from the PBS group. * Indicates significant difference from all the other groups. § Indicates significant difference from the LPS group. One symbol indicates p<0.05. Two symbols indicate p<0.01. Three symbols indicate p<0.001. The data on CpG are adapted from [11].

Acute (4 hours, 1 and 3 days) or chronic (7 and 14 days) toxicity of the adjuvants to the lungs were evaluated by measuring TNF-α (early local inflammation), total proteins, albumin (damage to the permeability of the pulmonary tissue) and the extracellular concentration of LDH (lung tissue damage) [28], [29]. CpG, LTB and the negative control PBS, had no effect on these biochemical parameters (Figs. 5A–D). MPLA induced an increase of total proteins and albumin at day 1 but these parameters returned to baseline values afterwards (Figs. 5A–B). MPLA also induced a significant LDH increase at day 3. Nevertheless, this increase was transient and lower than that generated by LPS (Fig. 5C). LPS, the positive control, induced a peak of TNF-α production 4 hours after its pulmonary delivery followed by increased concentrations of total proteins, albumin and LDH at day 3 (Figs. 5A–D). Yet, all these parameters rapidly returned to baseline levels.

### Cellular Components Generated by the Pulmonary Delivery of Adjuvants

**Figure 6:**
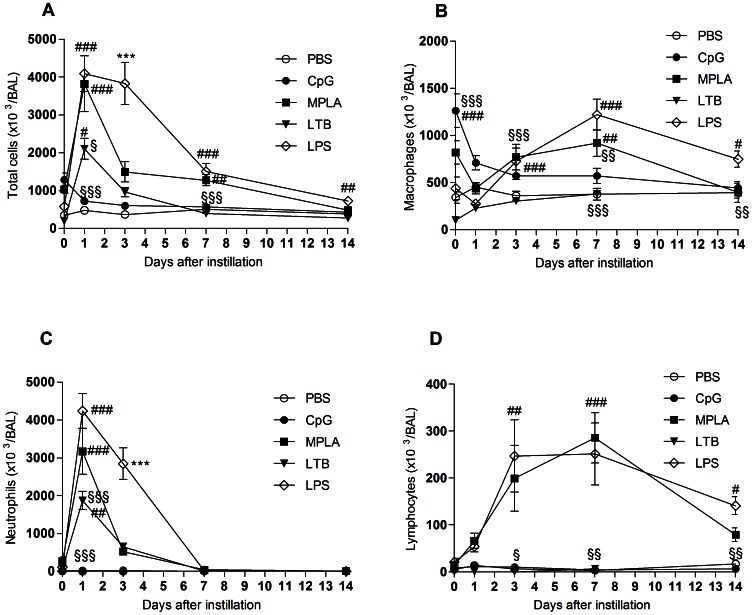
Effect of the adjuvants on inflammatory cellular components. A. Total cells. B. Macrophages. C. Neutrophils. D. Lymphocytes. # Indicates significant difference from the PBS group. * Indicates significant difference from all the other groups. § Indicates significant difference from the LPS group. One symbol indicates p<0.05. Two symbols indicate p<0.01. Three symbols indicate p<0.001. The data on CpG are adapted from [11].

BAL cell population was also analyzed following pulmonary delivery of the three adjuvants. Total cells increased 1 day after administration of MPLA, LTB and LPS. They subsequently decreased at day 3, except for LPS for which they decreased only at day 7 (Fig. 6A). This increase in total cells paralleled the increase in neutrophils (Fig. 6C). Lymphocytes significantly increased from day 1 up to day 7 following pulmonary delivery of LPS and MPLA (Fig. 6D). CpG, as the negative control PBS, did not affect total cells in BAL (Fig. 6A). CpG only transiently increased macrophages 4 hours and 1 day after administration (Fig. 6B).

### Protective Efficacy of the Subunit Vaccines

**Figure 7:**
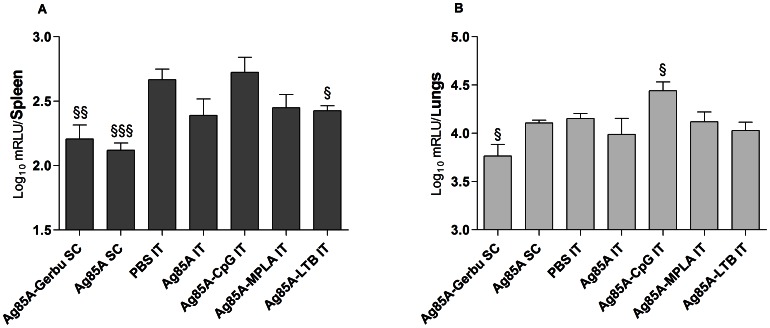
Protective efficacy of subunit vaccines. Replication of luminescent *M. tuberculosis* H37Rv four weeks after an intratracheal challenge in A. spleen and B. lungs. Luminometry results were expressed in milli relative units (mRLU) per total organ and were converted to log_10_ values. SC, subcutaneous injection; IT, intratracheal instillation. § Indicates significant difference from the PBS group. Student’s *t-*test: one symbol indicates p<0.05; two symbols indicate p<0.01; three symbols indicate p<0.001.

Subcutaneous vaccination with Ag85A in Gerbu generated significant protection both in spleen (Fig. 7A) and in lungs (Fig. 7B) against a virulent challenge with *M. tuberculosis* H37Rv, as compared to non-vaccinated mice. The log_10_ mRLU decrease in spleen and lungs was 0.48 and 0.37, respectively. Deep lung vaccination with Ag85A with or without MPLA or LTB tended to decrease bacterial counts both in spleen and lungs, as compared to non-vaccinated mice, with a significant decrease of 0.26 mRLU in the spleen for the LTB adjuvant (Fig. 7A). By contrast, CpG tended to increase the log_10_ mRLU in spleen and lungs with a significant increase of 0.31 mRLU in lungs, as compared to non-vaccinated mice (Fig. 7B). Bacterial counts in control groups treated with CpG, MPLA or LTB alone in the deep lungs were not different from bacterial counts in PBS-treated mice (data not shown).

## Discussion

The aim of this study was to assess immune responses to a subunit vaccine composed of Ag85A of *M. tuberculosis* and one of the following adjuvants CpG, MPLA or LTB after administration to the deep lungs in BALB/c mice. The three molecules showed a clear adjuvant effect reflected by higher IgG titers as compared to those induced by lung administration of the Ag85A alone (Fig. 2). In addition, each adjuvant induced a specific type of T cell response, as illustrated by cytokine production by *in vitro* restimulated spleen cells. CpG promoted a Th-1 type response (Fig. 3), MPLA induced a mixed Th-1 and Th-17 response (Figs. 3–4), whereas LTB induced a Th-2 type response (Fig. 3). Protection against infection with *M. tuberculosis* involves different cellular mechanisms [30], [31]. Multifunctional CD4+ Th-1 cells that coproduce IFN-γ, TNF-α and IL-2 are strongly associated with protection against *M. tuberculosis* infection. These cytokines synergize to activate intracellular microbicidal activities of alveolar macrophages. The developed Th-1 immune response is sufficient to contain bacilli inside granuloma and prevent further spread [31]. On the other hand, CD4+ T cells expressing IL-17 in the early phase of immunization contribute to the adaptive immune responses to mycobacteria by triggering expression of chemokines in the lung, which in turn may mediate recruitment of Th-1 cells to the airways. By contrast, a Th-2 response is generally associated with a poor protection against intracellular mycobacteria.

Based on our observations, MPLA and CpG represent promising adjuvants for pulmonary vaccination with Ag85A. These settings induced a better response than non-adjuvanted subcutaneous vaccination based on IgG levels, on the IgG2a/IgG1 ratio, which reflects the Th-1 response, and on cytokine production by spleen cells (Figs. 2 and 3). This is in line with our previous results on pulmonary vaccination using CpG [11]. Nevertheless, among the different conditions tested here, parenteral immunization with Gerbu adjuvant, used as a positive control, generated the highest immune response, in terms of immunoglobulin titers, IL-2 production by spleen cells and protective efficacy against an intratracheal *M. tuberculosis* challenge. Gerbu adjuvant has been developed as a more biotolerable alternative to complete Freund adjuvant [24] and is currently used for immunization of breeding animals but not of humans. By contrast, CpG oligonucleotides and MPLA have been developed for clinical applications [19]–[21].

In this study, vaccination-induced Th-1 responses were assessed both *in vitro* by restimulating spleen cells with the antigen and *in vivo* by pulmonary re-instillation with the same antigen (Figs. 3 and 4). Interestingly, splenocytes produced high and comparable levels of IFN-γ following *in vitro* restimulation with Ag85A in the group vaccinated subcutaneously with Gerbu and in the groups vaccinated in the lungs with CpG or MPLA (Fig. 3). However, following *in vivo* restimulation of the lungs with Ag85A, the group vaccinated subcutaneously with Gerbu largely outweighed the other groups in its IFN-γ production and expression of Iigp-1, an IFN-γ responsive biomarker (Fig. 4). These results correlated well with the protection results where mice vaccinated subcutaneously with Gerbu were protected against virulent *M. tuberculosis* challenge while mice vaccinated in the lungs were less protected (Fig. 7). This indicates that *in vitro* restimulation of memory T cells in the spleen is not predictive of *in vivo* restimulation of memory T cells in the lungs. Different hypotheses can be raised to explain this discrepancy. Some vaccinations might induce better local than systemic immune responses, but in our case, one would have expected a higher IFN-γ production of lung-vaccinated mice. Following airway deposition of Ag85A, systemically-activated T cells are rapidly mobilized into the airway lumen and recruited T cells produce IFN-γ [32]. This recruitment could explain the strong IFN-γ production in the lungs observed in the group vaccinated subcutaneously with Gerbu and subsequently restimulated with Ag85A in the lungs. In addition, activation of Th-1 memory T cells might have different requirement *in vivo* and *in vitro*. Antigen presentation and more specifically the type of antigen-presenting cells could be a critical issue as, *in vitro* T lymphocytes are activated by spleen antigen-presenting cells exposed to large quantities of antigen, while, *in vivo*, antigen presentation in the lung could be provided by functionally different cell types. Incidentally, mice vaccinated with Gerbu showed a higher IL-2 production (Fig. 3c), which might be critical *in vivo* for efficient activation and expansion of Th-1 lymphocytes, but less important *in vitro*.

Similarly, the observation that mice vaccinated with the LTB adjuvant showed Ag85A-specific IL-5 production in spleen contrasted with the fact that pulmonary restimulation with the same antigen did not lead to lung IL-5 production. In contrast, IL-17 production induced by the MPLA adjuvant could be detected both in spleen cells and in Ag85A-challenged lungs (Figs. 3 and 4). These observations could reflect *in vivo* interference between T helper subsets and more specifically inhibition by Ag85A-induced IFN-γ of lung Th-2 but not Th-17 activation, as described in similar models [33]. Nevertheless, taken together, these observations indicate that *in vitro* cytokine production by spleen cells and lung *in vivo* responses represent complementary pieces of information to assess the type of immune response induced by such vaccinations.

Delivery of TB vaccines to the respiratory tract has been shown critically advantageous over systemic administration for protection against TB [34]–[37]. BCG prepared as a spray-dried powder aerosol and delivered to the lungs in guinea pigs reduced bacterial burden and lung pathology relative to animals immunized with the standard subcutaneous BCG [36]. Intranasal delivery of a fusion protein consisting of Ag85B and the early secreted antigenic target 6 (ESAT6) and added in LTK63, an *Escherichia coli* heat-labile enterotoxin mutant, led to long lasting protection against TB, equivalent to that observed with BCG, as well as boosted prior BCG-stimulated immunity [37]. The rapid recruitment or persistence of antigen-specific T cells in the airway lumen probably holds the key to robust immune protection against TB [32], [38], [39]. Delivery of subunit TB vaccines to the respiratory tract elicited high numbers of Ag-specific CD4 and CD8 T cells in the airway lumen that were capable of IFN-γ production and cytolytic activities, while parenteral immunization failed to induce any T cell response in the airway lumen but rather led to activation of T cells in the spleen [32]. Upon exposure to *M. tuberculosis*, systemically-activated T cells cannot be rapidly mobilized into the airway lumen, as they are following deposition of soluble mycobacterial antigen [38]. This was in line with our protection study, where Ag85A administered alone subcutaneously protected mice in spleen but did not protect mice in the lungs against a challenge with virulent *M. tuberculosis* (Fig. 7). However, protection was restored in the lungs by adding the potent adjuvant Gerbu (Fig. 7B). The slow mobilization of T cells locally following exposure to *M. tuberculosis* may be explained in one hand by the ability of the bacillus to evade host immune attacks in order to prevent complete eradication. *M. tuberculosis* creates a protected niche for itself by modulating events in endosomal/phagosomal maturation. On the other hand, it may be explained by the fact that *M. tuberculosis* is a slow growing pathogen that delays the initiation of adaptive T cell immunity [40], [41].

Although the subunit vaccines composed of Ag85A and CpG or MPLA showed favorable mucosal and systemic immune responses, these did not translate in improved protection against a virulent challenge with *M. tuberculosis* H37Rv, as compared to Ag85A alone (Fig. 7). Although CpG induced high levels of IFN-γ in the spleen, it increased bacterial counts in the lungs. This observation correlates with CpG-induced increase in IL-10, a potent anti-inflammatory cytokine, in the lungs (Fig. 4D). It highlights the importance of cytokine production in the lungs to induce protection. As compared with the study using Ag85B-ESAT6 adjuvanted in LTK63 [37], our subunit vaccines were dosed five-times less in antigen and twice less in adjuvant. Therefore, our vaccination protocol could be optimized by using higher doses of antigen or the combined use of CpG and MPLA.

Safety of the adjuvants was also assessed in order to compare immunization efficacy and safety of these three clinically relevant adjuvants following pulmonary delivery. The adjuvants used in our study either did not induce any toxicity in the mouse lungs or, induced quickly resolved alterations to the pulmonary tissue (Figs. 5 and 6). CpG did not alter any of the biochemical and cellular inflammation markers measured in BAL after pulmonary delivery. LTB only induced a transient influx of neutrophils within the airway lumen 1 day post-delivery. MPLA transiently increased the permeability of the alveolo-capillary barrier 1 day post-delivery, generated a brief and slight damage to the lung tissue 3 days post-delivery, induced a transient influx of neutrophils within the airway lumen 1 day post-delivery and increased lymphocytes from day 1 up to day 7 post-delivery. Yet, the inflammation caused by MPLA was lower than that induced by LPS, probably due to the additional My88 signalling pathway activated by LPS [16]. Because the lungs are not connected to the brain as is the nasal mucosa through the olfactory epithelium, pulmonary vaccination does not present the risk of toxicity to the central nervous system, as intranasal vaccination does. In 2008, a phase I clinical trial of the nasal subunit vaccine composed of the fusion protein Ag85B-ESAT6 and the adjuvant LTK63 was terminated after a case of transient Bell’s Palsy caused by penetration of the LTK63 adjuvant into the brain [42].

### Conclusion

Pulmonary delivery of Ag85A admixed with CpG or MPLA generated a well polarized Th-1 immunity needed to protect against TB. In addition, MPLA generated a Th-17 polarized immunity in the lungs essential during the early phase of mycobacterial containment. CpG did not cause any inflammation in the lungs, while MPLA induced a quickly resolved inflammation. However, pulmonary administration of the adjuvants did not improve the protection provided by Ag85A alone against a virulent challenge with *M. tuberculosis*, pointing out the need for further optimization of the vaccination protocol. For this purpose, combining CpG and MPLA might be particularly promising because CpG could enhance the adjuvant effect of MPLA without increasing its toxicity.

## Supporting Information

Figure S1 **IL-17A production in BALs.** BAL samples were collected 24 h after *in vivo* restimulation with Ag85A or OVA. BAL samples were concentrated 2.5 times to measure IL-17A local production. Limit of detection = 26 pg/ml. SC, subcutaneous injection; IT, intratracheal instillation. § Indicates significant difference from the PBS group. # Indicates significant difference from the group vaccinated in the deep lung with Ag85A alone. One symbol indicates p<0.05.
(TIF)

## References

[1] World Health Organization (2010) Tuberculosis - Fact sheet n°104.

[2] Roche PW, Triccas JA, Winter N (1995) BCG vaccination against tuberculosis: past disappointments and future hopes. Trends Microbiol 3: 397–401. 8564359 10.1016/s0966-842x(00)88986-6

[3] World Health Organization (2006) The Stop TB Strategy.

[4] Stop TB Partnership (2009) Tuberculosis vaccine candidates.

[5] Scriba TJ, Tameris M, Mansoor N, Smit E, van der Merwe L, et al (2011) Dose-finding study of the novel tuberculosis vaccine, MVA85A, in healthy BCG-vaccinated infants. J Infect Dis 203: 1832–1843. 21606542 10.1093/infdis/jir195

[6] Elamin AA, Stehr M, Oehlmann W, Singh M (2009) The mycolyltransferase 85A, a putative drug target of Mycobacterium tuberculosis: development of a novel assay and quantification of glycolipid-status of the mycobacterial cell wall. J Microbiol Methods 79: 358–363. 19857528 10.1016/j.mimet.2009.10.010

[7] Romano M, Huygen K (2012) An update on vaccines for tuberculosis - there is more to it than just waning of BCG efficacy with time. Expert Opin Biol Ther 12: 1601–1610. 22957516 10.1517/14712598.2012.721768

[8] Lu D, Hickey AJ (2007) Pulmonary vaccine delivery. Expert Rev Vaccines 6: 213–226. 17408371 10.1586/14760584.6.2.213

[9] Holt PG, Strickland DH, Wikstrom ME, Jahnsen FL (2008) Regulation of immunological homeostasis in the respiratory tract. Nat Rev Immunol 8: 142–152. 18204469 10.1038/nri2236

[10] Minne A, Louahed J, Mehauden S, Baras B, Renauld JC, et al (2007) The delivery site of a monovalent influenza vaccine within the respiratory tract impacts on the immune response. Immunology 122: 316–325. 17521369 10.1111/j.1365-2567.2007.02641.x PMC2266027

[11] Todoroff J, Ucakar B, Inglese M, Vandermarliere S, Fillee C, et al (2012) Targeting the deep lungs, Poloxamer 407 and a CpG oligonucleotide optimize immune responses to Mycobacterium tuberculosis antigen 85A following pulmonary delivery. European Journal of Pharmaceutics and Biopharmaceutics doi: 10.1016/j.ejpb.2012.11.020. 10.1016/j.ejpb.2012.11.020 23238272

[12] Baldridge JR, Yorgensen Y, Ward JR, Ulrich JT (2000) Monophosphoryl lipid A enhances mucosal and systemic immunity to vaccine antigens following intranasal administration. Vaccine 18: 2416–2425. 10738099 10.1016/s0264-410x(99)00572-1

[13] Fingerut E, Gutter B, Goldway M, Eliahoo D, Pitcovski J (2006) B subunit of E. coli enterotoxin as adjuvant and carrier in oral and skin vaccination. Vet Immunol Immunopathol 112: 253–263. 16701905 10.1016/j.vetimm.2006.03.005

[14] Krieg AM (2000) Immune effects and mechanisms of action of CpG motifs. Vaccine 19: 618–622. 11090712 10.1016/s0264-410x(00)00249-8

[15] Fischer G, Conceiçao F, Leite F, Moraes C, Ferreira L, et al (2010) Recombinant Escherichia coli heat-labile enterotoxin B subunit humoral adjuvant effect depends on dose and administration route. World Journal of Microbiology and Biotechnology 26: 489–495.

[16] Mata-Haro V, Cekic C, Martin M, Chilton PM, Casella CR, et al (2007) The vaccine adjuvant monophosphoryl lipid A as a TRIF-biased agonist of TLR4. Science 316: 1628–1632. 17569868 10.1126/science.1138963

[17] Krieg AM (2006) Therapeutic potential of Toll-like receptor 9 activation. Nat Rev Drug Discov 5: 471–484. 16763660 10.1038/nrd2059

[18] da Hora VP, Conceicao FR, Dellagostin OA, Doolan DL (2010) Non-toxic derivatives of LT as potent adjuvants. Vaccine 29: 1538–1544. 21163247 10.1016/j.vaccine.2010.11.091

[19] Barry M, Cooper C (2007) Review of hepatitis B surface antigen-1018 ISS adjuvant-containing vaccine safety and efficacy. Expert Opin Biol Ther 7: 1731–1737. 17961095 10.1517/14712598.7.11.1731

[20] Casella CR, Mitchell TC (2008) Putting endotoxin to work for us: monophosphoryl lipid A as a safe and effective vaccine adjuvant. Cell Mol Life Sci 65: 3231–3240. 18668203 10.1007/s00018-008-8228-6 PMC2647720

[21] Stephenson I, Zambon MC, Rudin A, Colegate A, Podda A, et al (2006) Phase I evaluation of intranasal trivalent inactivated influenza vaccine with nontoxigenic Escherichia coli enterotoxin and novel biovector as mucosal adjuvants, using adult volunteers. J Virol 80: 4962–4970. 16641287 10.1128/JVI.80.10.4962-4970.2006 PMC1472052

[22] Gartner T, Baeten M, Otieno S, Revets H, De Baetselier P, et al (2007) Mucosal prime-boost vaccination for tuberculosis based on TLR triggering OprI lipoprotein from Pseudomonas aeruginosa fused to mycolyl-transferase Ag85A. Immunol Lett 111: 26–35. 17570535 10.1016/j.imlet.2007.04.010

[23] D’Souza S, Rosseels V, Romano M, Tanghe A, Denis O, et al (2003) Mapping of murine Th1 helper T-Cell epitopes of mycolyl transferases Ag85A, Ag85B, and Ag85C from Mycobacterium tuberculosis. Infect Immun 71: 483–493. 12496199 10.1128/IAI.71.1.483-493.2003 PMC143283

[24] Grubhofer N (1995) An adjuvant formulation based on N-acetylglucosaminyl-N-acetylmuramyl-L-alanyl-D-isoglutamine with dimethyldioctadecylammonium chloride and zinc-L-proline complex as synergists. Immunol Lett 44: 19–24. 7721338 10.1016/0165-2478(94)00188-w

[25] Snewin VA, Gares MP, Gaora PO, Hasan Z, Brown IN, et al (1999) Assessment of immunity to mycobacterial infection with luciferase reporter constructs. Infect Immun 67: 4586–4593. 10456904 10.1128/iai.67.9.4586-4593.1999 PMC96782

[26] Romano M, Roupie V, Wang XM, Denis O, Jurion F, et al (2006) Immunogenicity and protective efficacy of tuberculosis DNA vaccines combining mycolyl-transferase Ag85A and phosphate transport receptor PstS-3. Immunology 118: 321–332. 16827893 10.1111/j.1365-2567.2006.02373.x PMC1782306

[27] Cooper AM, Torrado E (2012) Protection versus pathology in tuberculosis: recent insights. Curr Opin Immunol 24: 431–437. 22613092 10.1016/j.coi.2012.04.008 PMC3423558

[28] Nicod LP (1999) Pulmonary defence mechanisms. Respiration 66: 2–11. 9973683 10.1159/000029329

[29] Drent M, Cobben NA, Henderson RF, Wouters EF, van Dieijen-Visser M (1996) Usefulness of lactate dehydrogenase and its isoenzymes as indicators of lung damage or inflammation. Eur Respir J 9: 1736–1742. 8866602 10.1183/09031936.96.09081736

[30] Boom WH (1996) The role of T-cell subsets in Mycobacterium tuberculosis infection. Infect Agents Dis 5: 73–81. 8721044

[31] Ottenhoff TH (2012) New pathways of protective and pathological host defense to mycobacteria. Trends Microbiol 20: 419–428. 22784857 10.1016/j.tim.2012.06.002

[32] Santosuosso M, Zhang X, McCormick S, Wang J, Hitt M, et al (2005) Mechanisms of mucosal and parenteral tuberculosis vaccinations: adenoviral-based mucosal immunization preferentially elicits sustained accumulation of immune protective CD4 and CD8 T cells within the airway lumen. J Immunol 174: 7986–7994. 15944305 10.4049/jimmunol.174.12.7986

[33] Lemaire MM, Dumoutier L, Warnier G, Uyttenhove C, Van Snick J, et al (2011) Dual TCR expression biases lung inflammation in DO11.10 transgenic mice and promotes neutrophilia via microbiota-induced Th17 differentiation. J Immunol 187: 3530–3537. 21859957 10.4049/jimmunol.1101720

[34] Wang J, Thorson L, Stokes RW, Santosuosso M, Huygen K, et al (2004) Single mucosal, but not parenteral, immunization with recombinant adenoviral-based vaccine provides potent protection from pulmonary tuberculosis. J Immunol 173: 6357–6365. 15528375 10.4049/jimmunol.173.10.6357

[35] Goonetilleke NP, McShane H, Hannan CM, Anderson RJ, Brookes RH, et al (2003) Enhanced immunogenicity and protective efficacy against Mycobacterium tuberculosis of bacille Calmette-Guerin vaccine using mucosal administration and boosting with a recombinant modified vaccinia virus Ankara. J Immunol 171: 1602–1609. 12874255 10.4049/jimmunol.171.3.1602

[36] Garcia-Contreras L, Wong YL, Muttil P, Padilla D, Sadoff J, et al (2008) Immunization by a bacterial aerosol. Proc Natl Acad Sci U S A 105: 4656–4660. 18344320 10.1073/pnas.0800043105 PMC2290758

[37] Dietrich J, Andersen C, Rappuoli R, Doherty TM, Jensen CG, et al (2006) Mucosal administration of Ag85B-ESAT-6 protects against infection with Mycobacterium tuberculosis and boosts prior bacillus Calmette-Guerin immunity. J Immunol 177: 6353–6360. 17056566 10.4049/jimmunol.177.9.6353

[38] Jeyanathan M, Mu J, Kugathasan K, Zhang X, Damjanovic D, et al (2008) Airway delivery of soluble mycobacterial antigens restores protective mucosal immunity by single intramuscular plasmid DNA tuberculosis vaccination: role of proinflammatory signals in the lung. J Immunol 181: 5618–5626. 18832720 10.4049/jimmunol.181.8.5618

[39] Santosuosso M, McCormick S, Roediger E, Zhang X, Zganiacz A, et al (2007) Mucosal luminal manipulation of T cell geography switches on protective efficacy by otherwise ineffective parenteral genetic immunization. J Immunol 178: 2387–2395. 17277145 10.4049/jimmunol.178.4.2387

[40] Banaiee N, Kincaid EZ, Buchwald U, Jacobs WR Jr, Ernst JD (2006) Potent inhibition of macrophage responses to IFN-gamma by live virulent Mycobacterium tuberculosis is independent of mature mycobacterial lipoproteins but dependent on TLR2. J Immunol 176: 3019–3027. 16493060 10.4049/jimmunol.176.5.3019

[41] Sundaramurthy V, Pieters J (2007) Interactions of pathogenic mycobacteria with host macrophages. Microbes Infect 9: 1671–1679. 18023233 10.1016/j.micinf.2007.09.007

[42] Lewis DJ, Huo Z, Barnett S, Kromann I, Giemza R, et al (2009) Transient facial nerve paralysis (Bell’s palsy) following intranasal delivery of a genetically detoxified mutant of Escherichia coli heat labile toxin. PLoS One 4: e6999. 19756141 10.1371/journal.pone.0006999 PMC2737308

