# Supplementary figures and images for: Mucosal and Systemic Immune Responses to Mycobacterium tuberculosis Antigen 85A following Its Co-Delivery with CpG, MPLA or LTB to the Lungs in Mice
Source: PLoS One. 2013 May 10;8(5):e63344. doi: 10.1371/journal.pone.0063344 (PMC3651129; doi:10.1371/journal.pone.0063344)

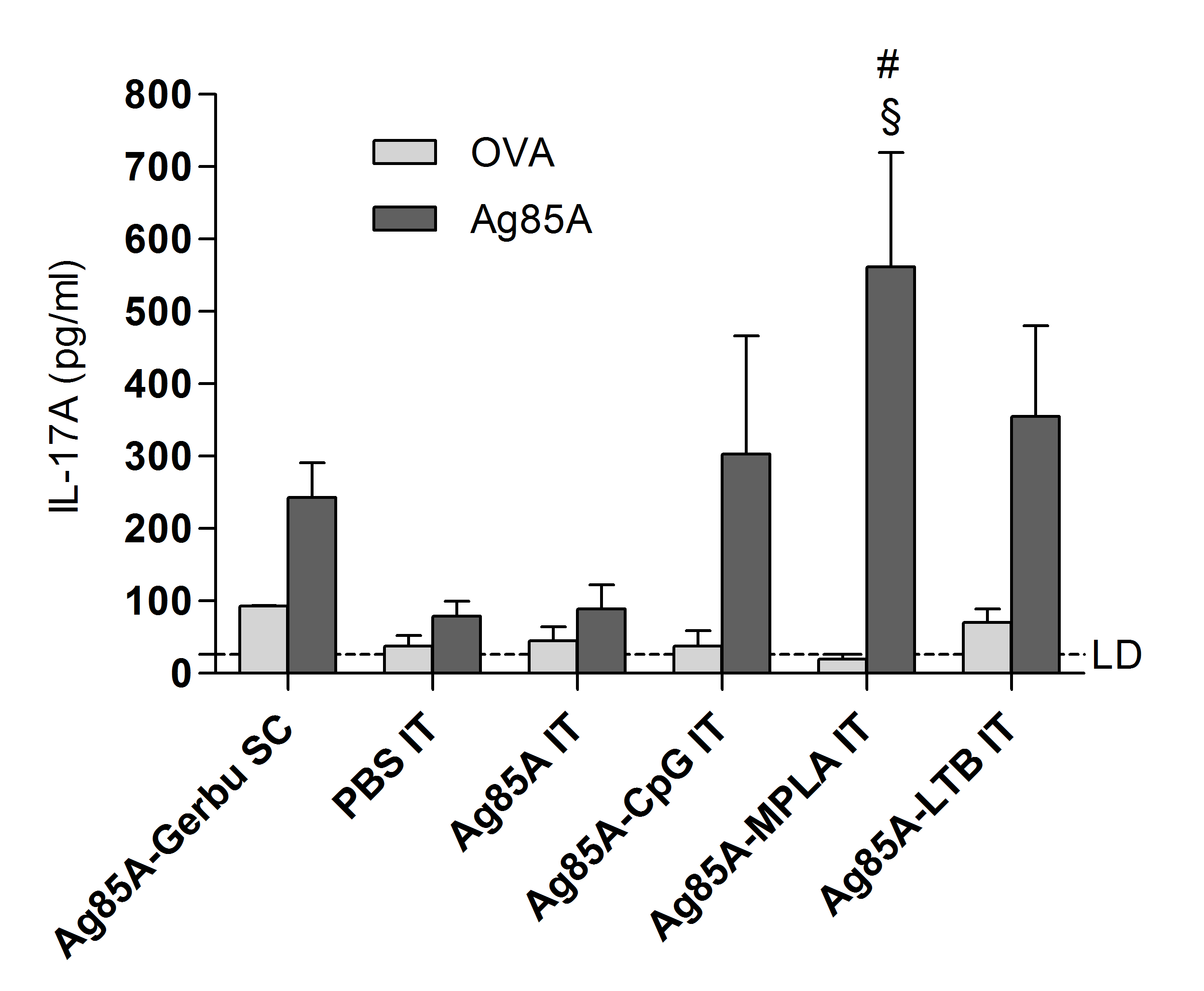

Supplement: Figure S1 — IL-17A production in BALs. BAL samples were collected 24 h after in vivo restimulation with Ag85A or OVA. BAL samples were concentrated 2.5 times to measure IL-17A local production. Limit of detection = 26 pg/ml. SC, subcutaneous injection; IT, intratracheal instillation. § Indicates significant difference from the PBS group. # Indicates significant difference from the group vaccinated in the deep lung with Ag85A alone. One symbol indicates p<0.05. (TIF) [file pone.0063344.s001.tif]
